# Effects of Rhizobacteria Strains on Plant Growth Promotion in Tomatoes (*Solanum lycopersicum*)

**DOI:** 10.3390/plants13233280

**Published:** 2024-11-22

**Authors:** Eduardo Hernández-Amador, David Tomás Montesdeoca-Flores, Néstor Abreu-Acosta, Juan Cristo Luis-Jorge

**Affiliations:** 1 Department of Botany, Ecology and Plant Physiology, Area of Plant Physiology, Science Faculty, University of La Laguna, Avenida Astrofísico Francisco Sánchez s/n, 38200 San Cristóbal de La Laguna, Tenerife, Spain; dmontesd@ull.edu.es; 2 Nertalab S.L., C. José Rodríguez Moure 4, 38008 Santa Cruz de Tenerife, Tenerife, Spain; gerencia@nertalab.es

**Keywords:** plant-growth-promoting rhizobacteria, PGPR, tomato, *biofertilizer*, *Solanum lycopersicum*

## Abstract

Numerous factors, such as soil fertility, climatic conditions, human activity, pests, and diseases, limit agricultural yields. Pesticides and fertilizers have become indispensable tools to satisfy the global food demand. However, its adverse environmental effects have led to the search for more sustainable and ethical techniques. Biofertilizers and biopesticides based on plant- growth-promoting rhizobacteria (PGPRs) are efficient and ecological treatments that promote plant growth and protection against pathogens and abiotic stresses. In this study, twelve rhizobacterial strains with plant-growth-promoting attributes were selected to evaluate their plant-growth-promoting effect on tomato plants (*Solanum lycopersicum* L. var Robin). Soil inoculation with these strains resulted in a significant increase in shoot length, up to 50% when compared with control plants. Regarding fresh biomass, rhizobacterial treatments significantly improved seedlings’ fresh aerial weight with a maximum increase of 77%. Root biomass also demonstrated a substantial improvement, yielding 62.26% greater fresh root weight compared to the control. Finally, dry root weights exhibited the most remarkable enhancements, with values between 49 and 124%, when compared to the control plants. Concerning the nutritional status, the strains inoculation increased the macronutrients and micronutrients content in the aerial and root parts of the plants. All these findings suggest that rhizobacteria from different ecosystems and agriculture soils of the Canary Islands could be used as fertilizer inoculants to increase crop yield and promote more sustainable practices in modern agriculture.

## 1. Introduction

Agriculture is a fundamental activity for humankind, providing essential food for human and animal nutrition. An environmentally friendly approach to agriculture is needed to ensure food security and address global challenges such as climate change and biodiversity loss [1]. The use of synthetic fertilizers and pesticides has led to a series of adverse effects on soil health, impacting its microbiota and fertility, while also being a source of pollution [2]. In recent decades, the scientific community has focused on reversing this trend, exploring new biological techniques that are more ethical and sustainable for humans and the environment [3]. Among these novel approaches, the study of the rhizosphere and the use of rhizobacteria have emerged as key areas due to their beneficial relationship with plants, which is facilitated by direct and indirect mechanisms [4]. Consequently, rhizosphere management is proposed as a relevant area for agrobiotechnology to increase yield and agricultural production while minimizing controlling the use of limited, contaminant, and costly resources [5].

Rhizobacterial microorganisms play a crucial role in plant growth and development by influencing nutrient availability, soil health, and resistance to biotic and abiotic stresses through a variety of mechanisms that allow them to interact favorably with plants [6,7]. These rhizobacteria are called plant-growth-promoting rhizobacteria (PGPRs). There is research reporting the use of microorganisms belonging to a large number of genera, including *Achromobacter*, *Agrobacterium*, *Alcaligenes*, *Arthrobacter*, *Azospirillum*, *Azotobacter*, *Bacillus*, *Burkholderia*, *Enterobacter*, *Flavobacterium*, *Frankia*, *Klebsiella*, *Micrococcus*, *Pseudomonas*, and *Serratia*, among others [4,8,9]. The mechanisms employed by PGPR are classified into direct and indirect mechanisms. Direct mechanisms include those biological processes related to nutrient mobilization and acquisition [10,11,12]. Rhizobacteria play a fundamental role in nitrogen fixation thanks to the action of the enzyme nitrogenase, which transforms atmospheric nitrogen into ammonium, a compound accessible to plant roots [13]. On the other hand, some rhizobacteria can solubilize phosphorus, transforming its insoluble forms into soluble organic forms. These bacteria have the enzyme phosphatase, as well as the release of organic acids that solubilize phosphorus from different sources, increasing its availability and accessibility to plants [14]. Potassium is another macronutrient found in many chemical forms that are not assimilable by plants. Therefore, PGPRs appear on the horizon as a biological tool capable of potassium solubilization. The mechanism employed by these bacterial strains is based on the release of organic acids that transform insoluble potassium from minerals into its assimilable form to plants [15,16]. Similarly, they produce phytohormones that directly influence plant development, such as auxins, cytokinins, gibberellins, abscisic acid, or ethylene [16,17,18,19]. They also improve iron availability through to the production of siderophores that act as chelating agents of this element and other metals [20,21].

On the other hand, during biotic and abiotic stress situations that alter plant physiology and metabolism, responses are activated by rhizobacteria to alleviate these phenomena. Under biotic stress conditions, rhizobacteria present several tools to manage the battle against pathogens and diseases [22]. Among these tools are lytic enzyme production, activation of the induced-resistance system, antibiotic production, biofilm formation, quorum sensing, volatile organic compounds (VOCs) production, or hydrogen cyanide production [3,23,24,25]. Abiotic stresses such as salinity, drought, extreme temperatures, and the presence of heavy metals have been increasing in recent years [19]. Thus, in this scenario, rhizobacteria appear as a strategy to mitigate and protect plants from these increasingly daily and periodic stresses. Rhizobacteria are involved in osmotic regulation, reduction in oxidative stress, enhancement of antioxidant activity, production of VOCs, phytohormones, and improved nutrient acquisition as pathways to combat salt stress [26,27]. During drought, rhizobacteria produce osmolytes, antioxidants, exopolysaccharides, VOCs, and changes in root structure that enhance tolerance to this stress [25,28]. Moreover, to cope with extreme temperatures, rhizobacteria enhance homeostasis and the antioxidant system, promoting the production of phytohormones, exopolysaccharides, and reducing ethylene levels through the enzyme 1-aminocyclopropane-1-carboxylate (ACC) deaminase [29,30,31]. In heavy metal accumulation, rhizobacteria favor plant growth thanks to the production of siderophores that sequester these harmful elements and by elevating the production of phytohormones, exopolysaccharides, and antioxidant enzymes [26,32].

As a result of the action of these mechanisms, there are numerous examples of beneficial effects of PGPR application on different crops. Arikan and Pirlak [33] demonstrated an improvement in the plant growth of sour cherry when inoculated alone or in combination with *Bacillus mycoides* T8 and *Bacillus subtilus* OSU-142 strains. On the other hand, the use of rhizobacteria isolated from rice, wheat, and maize rhizospheres (*Bacillus altitudinis*, *Rhizobium daejeonense*, *Pseudomonas monteilli*, *Enterobacter cloacae,* and *Bacillus pumilus*) improved plant development when tested in rice plants [34]. In a trial with potatoes treated with rhizobacteria isolated from their own rhizosphere (*Pseudomonas* sp., *Azospirillum* sp., *Enterobacter* sp., and *Rhizobium* sp.), plant height parameters, total fresh and dry weights, and plant N content improved [35]. Another example of the benefits of PGPR inoculation was reproduced by Pérez-García et al. [36] on cucumber seeds. *Acinetobacter radioresistens* (KBENdo3P1), *Pseudomonas paralactis* (KBENdo6P7), and *Bacillus cereus* (KBENdo4P6) strains promoted a significant response in seed germination vigor, plumules and roots development, and the synthesis of non-enzymatic antioxidant compounds.

However, several gaps exist in using PGPRs as biofertilizers and biocontrol agents. Despite decades of study, the molecular mechanisms involved in plant–bacteria interactions are still not fully understood [37]. Another area of improvement of rhizobacteria is their performance under field conditions, as some cannot meet the expectations achieved in the laboratory due to climatic conditions, soil physicochemical properties, the crop itself, and the already-existing microbiome [38]. Therefore, it is necessary to continue the search for new rhizobacteria that do not alter their performance once they leave the laboratory, trying to find the best possible formulations. In addition, farm acceptance is still a pending issue. It is necessary to illustrate the benefits of this fertilization technique, to search for easy application methods that do not complicate the work of producers, and to find a reasonable cost that justifies its use compared to traditional methods [9]. Moreover, there are limitations regarding legislation on PGPR-based biofertilizers [39]. The registration and use of possible biopreparations are uncertain, while awaiting management of these products in the short and medium term is essential to legally define their status, use, and commercialization.

One of the crops with an extensive number of studies on the application of rhizobacteria is tomato. Several studies used strains such as *Arthrobacter* sp., *Bacillus cenopacia*, *Bacillus licheniformis*, *Bacillus thuringiensis*, *Enterobacter* sp., *Proteus mirabilis*, and *Pseudomonas* sp. to improve tomato plant growth [40,41,42,43,44]. Tomato is a product with high commercial value due to its nutritional content, flavor, and health benefits [45]. These characteristics promote its production and consumption, making it one of the most important crops in the world, producing 189 million tons with more than five million hectares being cultivated [46,47]. Tomatoes and bananas have been the most used crops since the 19th century in the Canary Islands. Today, there is still a concentrated production in Tenerife and Gran Canaria that satisfies local needs and exports. However, the economic factor is one of the limiting reasons for its production in the archipelago, as it has to deal with strong competition from other markets with lower costs that have been gaining market share [48]. 

In this way, the use of PGPR-based biofertilizers can become a way to reduce the environmental impact of chemical fertilizers and at the same time enhance crop yields [49]. In this context, this study aimed to evaluate the effect of rhizobacteria from various ecosystems of the Canary Islands in promoting plant growth in tomato plants, something that has not been performed previously in the archipelago. The application of rhizobacteria is often connected to the isolation of specific strains found in the rhizosphere of targeted crops. This approach ensures the use of microorganisms that are already adapted to the particular environmental conditions of the crop. On the contrary, many researchers choose to explore different ecosystems around the world to isolate new PGPRs [50]. Prospecting in these habitats can lead to finding new strains with suitable characteristics to combat abiotic or biotic stresses common in their areas of origin, allowing for crops to increase their performance. For this reason, it was decided to take advantage of the environmental diversity of the Canary Islands, which is made up of five areas—ranging from the coast to the summit: coastal scrubland, dry sclerophyllous forests, humid evergreen forests, pine forests, and high mountain dry forest—to carry out isolations of rhizobacteria and discover the effects of strains of such varied origin in the promotion of tomato plant growth. In this context, this study aimed to evaluate the impact of rhizobacteria strains on plant growth promotion in tomato plants. Three types of analysis were performed to achieve this objective. The first, a non-destructive analysis, focused on an assessment of physiological parameters, including the measurement of photosynthesis rate and stomatal conductance, among others. The second analysis was biometric and focused on plant length and weight. Finally, a chemical analysis was performed to determine the content of total antioxidants, phenols, pigments, and the nutritional content of the tomato plants.

## 2. Materials and Methods

### 2.1. Soil

This study was initiated in pots with soil taken from the Higher Polytechnic School of Engineering, University of La Laguna, Canary Islands, Spain (28°28′47.5″ N 16°19′08.9″ W; altitude 550 m above sea level). The soil was sieved through a 2 mm sieve and autoclaved in bags in three cycles at 121 °C for 30 min, allowing 24 h to pass between cycles. To determine its physicochemical characteristics before the tests, a soil sample was dried at room temperature, and the following parameters were analyzed: pH; electrical conductivity in the saturation extract; % of saturation, organic matter; Olsen’s phosphorus; and assimilable Ca, Mg, Na, and K [51].

### 2.2. Rhizobacteria Strains

The bacterial strains used in this study were isolated from various agricultural soils and environments in the Canary Islands, Spain, as part of previous research [52]. Each strain was classified to its closest species based on sequence analysis and is currently preserved in the Nertalab S.L. strain collection. Twelve rhizobacteria with at least three plant growth promotion characteristics were selected based on their capacity in the mobilization and solubilization of nutrients such as N, P, and K, as well as the production of indole acetic acid (IAA) and siderophores. Thus, the strains selected are shown in Table 1.

### 2.3. Plant Growth Promotion Assays

The trial was carried out in 600 mL pots with autoclaved soil. After subjecting these pots to field capacity, tomato plants (*Solanum lycopersicum* L. var Robin) obtained from seedlings were planted in them one week after germination, at the cotyledon stage, and at the beginning of the appearance of the first true leaf. Fifteen plants were used for the control and treatments. Due to the availability of plants, the trial was carried out in three batches, the first one with *Providencia vermicola* 17, *Mitsuaria noduli* 19, *Bacillus siamensis* 1SEF, and *Bacillus velezensis* 4PIN. The second batch included *Pseudomonas plecoglossicida* 16, *Proteus mirabilis* 27, *Pantoea cypripedii* 32, and *Paenibacillus pabuli* 47. Finally, the third batch consisted of *Bacillus* sp. 6AB, *Bacillus megaterium* 8AB4, *Bacillus thuringiensis* 3GRA, and *Bacillus mycoides* 1CRN.

To prepare the inoculum, selected microorganisms were cultured from cryopreserved vials in tryptone soy agar media (TSA) for 24–48 h to obtain isolated colonies. Using an inoculating loop, bacterial culture was taken and resuspended in sterile 0.85% saline until a McFarland turbidity of 0.5 (10^8^ colony-forming unit (CFU) mL^−1^). In total, 100 μL of this suspension was taken, added to 5 mL of TSB, and incubated at 26 °C under continuous agitation for 24 h. After this, to inoculate each plant with 10^7^ CFU mL^−1^, the bacterial concentration of the cultures was measured spectrophotometrically (UV-6300PC, VWR International Europe BV, Leuven, Belgium). For this purpose, an absorbance standard line at 600 nm elaborated from a series of McFarland turbidity standards was used. The approximate equivalence between these standards and the cell density proposed by Gayathiri et al. [53] was considered. Once the concentration of the inoculum was known, we diluted them with Hoagland nutrient solution at 10^7^ CFU mL^−1^ for 20 mL [54]. 

The pots were randomly distributed in a culture chamber where the tests were carried out, which lasted 15 days. The light intensity conditions were measured with a quantum meter, generally 600–800 µmol m^−2^ s^−1^, the temperature was 25–27 °C, and the photoperiod was 16/8 h. Plants were irrigated at field capacity, and on day 1, plants were irrigated with 20 mL of Hoagland nutrient solution supplemented with 1 mL of bacterial inoculum. On days 2 and 3, irrigation was carried out with 30 mL and 40 mL of distilled water, respectively. From days 4 to 7, plants received 50 mL of distilled water daily. On day 8, a second application of 50 mL of the Hoagland nutrient solution and 1 mL of the bacterial inoculum was administered. From days 9 to 14, the plants were irrigated with 50 mL of distilled water daily, and non-destructive analyses were conducted. The experiment concluded on day 15.

### 2.4. Physiological, Morphological, Chemical, and Nutritional Analysis

Physiological, morphological, chemical, and nutritional analyses of the plants were carried out to evaluate the effect of the bacterial treatments on them. First, a non-destructive test was carried out to measure the parameters described below:

- Photosynthesis rate and stomatal conductance: An infrared gas analyzer (IRGA) (LCpro-SD, ADC BioScientific Ltd., Hoddesdon, UK) was used. The analysis was conducted in the culture chamber 2 h after turning on the lights. Healthy adult leaves from seven plants were randomly selected in triplicate for each treatment.
- Maximum photosystem II photochemical efficiency (Fv/Fm) and photosynthetic performance index: These were measured using a continuous excitation chlorophyll fluorimeter (Handy PEA, Hansatech Instruments Ltd., King´s Lynn, UK). In this case, the culture chamber remained in darkness for one hour before the measurement. Ten plants were randomly selected for each treatment.

The next analysis was the biometric analysis, which was performed as the final phase of the experiment. The plants were removed from the pots, and the substrate was discarded. The roots were cleaned thoroughly through several washes in water and then allowed to dry. The parameters that were analyzed are shown below:

- Aerial and root length: A ruler was used to measure them. The aerial and root parts were divided from the birth of the uppermost secondary root to establish both measurements.
- Aerial and root wet weight: As mentioned in the previous parameter, once the line separating the aerial and root parts was marked, they were cut and weighed separately on a scale.
- Aerial and root dry weight: The samples were dried in an oven at 65 °C for 3 days after measuring the fresh weight. After this time, the methodology described for the fresh weight was repeated.

Several fresh samples were separated during the destructive analysis to evaluate the antioxidant activity, phenols content, and photosynthetic pigments. For this purpose, the samples were frozen and stored at 80 °C. After that, the material was freeze-dried to remove moisture and ground to obtain a fine powder. This powder was stored at −20 °C until use. The extraction took place following the protocols established by Ruiz-Medina et al. [55]. First, 0.015 g of dry powder was resuspended in 2 mL of 80% methanol. Then, they were vortexed for 2 min to obtain a mixture of the components. Then, the contents were shaken for one and a half hours at 400 rpm on an orbital shaker and left to settle overnight. The following day, the supernatant was transferred to a test tube, while the resulting pellet was resuspended in 2 mL of solvent and vortexed for 30 s. Both supernatants from the extractions were combined, and the resulting extract was filtered through a syringe using a 0.45 μm Millipore filter to eliminate any particles or impurities. The samples were stored at −20 °C until needed. Extractions were conducted in triplicate in a randomized manner, using 3 out of 15 plants for each treatment to ensure reproducibility and minimize variability. The assays followed the instructions proposed by Ruiz-Medina et al. [55], which were as follows:

- Total antioxidant content: The 2,2-diphenyl-1-picrylhydrazyl (DPPH) free-radical-scavenging capacity of the leaf extracts was evaluated by adding 0.2 mL of the extract to 3.8 mL of a 0.25 mM methanolic DPPH solution. The mixture was vigorously shaken for 1 min and allowed to stand in the dark at room temperature for 30 min. The absorbance of the sample was measured using a UV–visible spectrophotometer (UV-6300PC, VWR International Europe BV, Leuven, Belgium) at a wavelength of 517 nm, with an ethanol blank serving as the reference. For the negative control, 80% methanol was employed in place of the extract. The free-radical-scavenging activity of the extracts was expressed in terms of Trolox equivalents. The results were calculated using a calibration curve and expressed in milligrams of Trolox equivalents per gram of dry weight. The linearity range of the calibration curve was 50 to 350 μg mL^−1^ (r = 0.998).
- Total phenolic content: Gallic acid was used as a standard. Thus, 50 μL extract was mixed with 1.5 mL of distilled water, 250 μL of Folin–Ciocalteu 2 N reagent solution, and 750 μL of 7% sodium carbonate. The obtained mixture was vortexed and incubated for 8 min at room temperature. Then, an additional 950 μL of distilled water was added and allowed to stand for 2 h at room temperature. Finally, absorbance was measured against a distilled water blank at a wavelength of 765 nm using a UV–visible spectrophotometer (UV-6300PC, VWR International Europe BV, Leuven, Belgium). Results were calculated and expressed in milligram gallic acid equivalents (mg GAE/g dry weight) using a previously established calibration curve with gallic acid concentrations from 10 to 400 μg mL^−1^ (r = 0.998).
- Pigment content: To prevent any acidic traces that could alter the pigment composition, 0.01 g of dry powder was combined with 2 mL of 100% acetone that had been previously saturated with calcium carbonate. After a 10 min centrifugation at 4 °C, the samples were filtered through a syringe with a 0.45 μm Millipore filter to remove impurities. Pigment quantification was conducted using a dual-beam spectrophotometer (UV-6300PC, VWR International Europe BV, Leuven, Belgium). To calculate chlorophyll a, chlorophyll b, and total carotenoids in mg g^−1^ dry weight, the equations given by Ruiz-Medina et al. [55] were used.

Finally, the nutritional content was evaluated thanks to the dry material of the root and aerial part of the plant. The dried samples were ground with an IKA M20 micromilling machine (IKA Werke GmbH & Co., Staufen im Breisgau, Germany) and placed in envelopes duly identified. They were kept in an oven at 105 °C for 5 h then transferred to a desiccator and finally weighed. Nitrogen was determined using the Kjeldahl method. A 0.1 g sample of dry material was taken for this purpose. For the nutrients Ca, Mg, Na, K, P, Cu, Fe, Mn, and Zn, 1 g of ground powder was taken from each leaf sample, which was dry-mineralized with 6N hydrochloric acid after calcination in a muffle furnace at 480 °C. The concentrations were determined using a Perkin Elmer ICP, model Optima 2000D (Perkin Elmer, Shelton, CT, USA). These characteristics were determined following the laboratory techniques described in the Official Methods of Analysis [51].

### 2.5. Statistical Analysis

Statistical analyses were performed with IBM SPSS Statistics for Windows, Version 26.0 (IBM, Armonk, NY, USA). First, for the biometric study, the normality, and homoscedasticity of each dataset were assessed using a Kolmogorov–Smirnov test and a Levene test, respectively. If these conditions were not met, non-parametric tests were used instead. In this case, a Kruskal–Wallis test was used, with the relevant pairwise comparisons, whose significance values were adjusted using a Bonferroni correction for several tests. On the other hand, homoscedasticity was assessed using the Levene’s test to analyze the differences between extracts. Normality was verified using a Shapiro–Wilk test, as the sample size in this case was less than 50.

## 3. Results

### 3.1. Soil Analysis

The results of the physicochemical analysis classified the soil as chemically adequate. The pH was alkaline, almost neutral, with a medium–low organic matter content and low salinity indexes. In addition, the potassium content was correct for assimilation, but the assimilable phosphorus content was slightly low (Table 2).

### 3.2. Physiological, Chemical, and Nutritional Analysis

**Figure 1:**
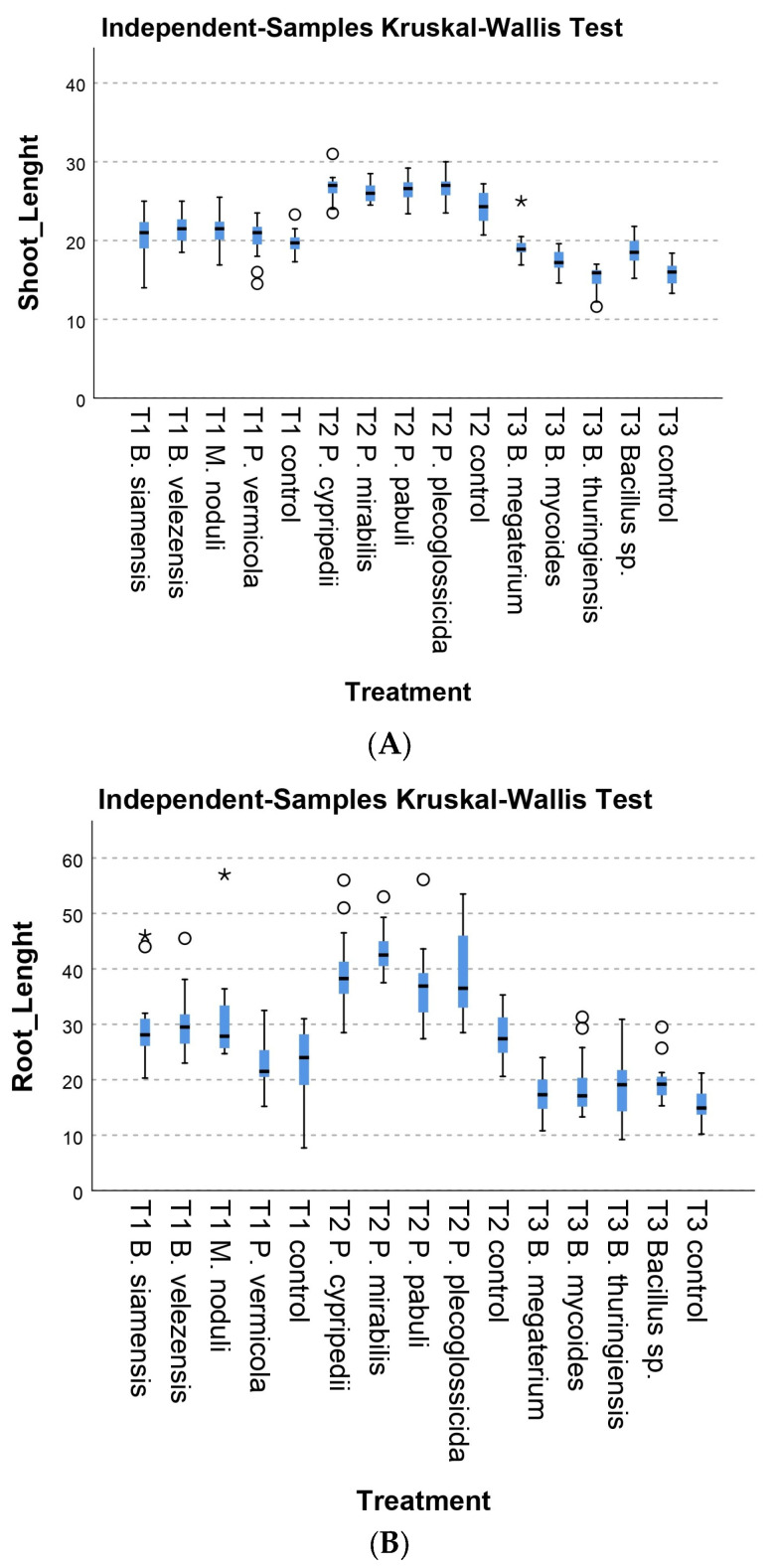

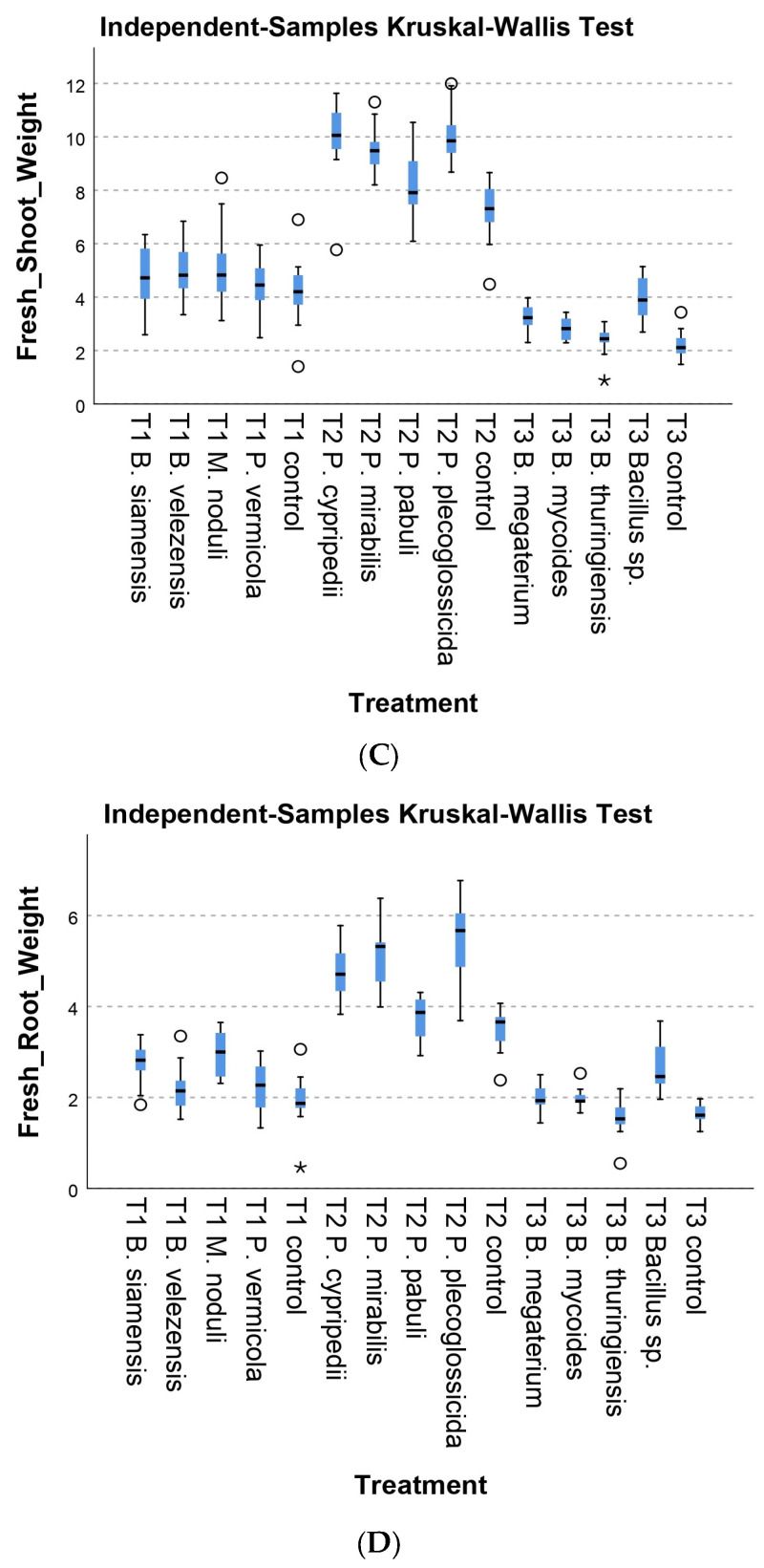

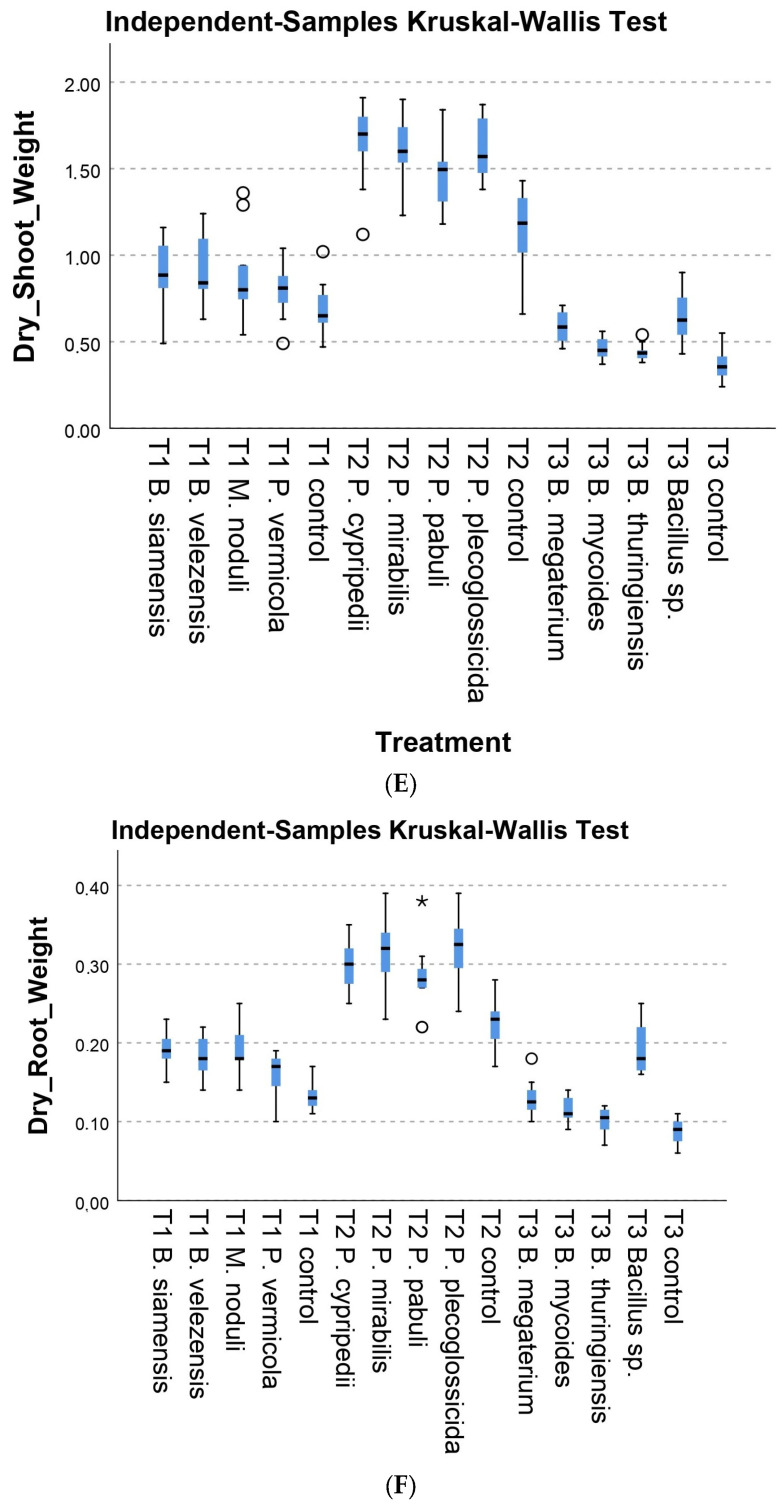
Kruskal–Wallis tests for the six morphological variables studied. Note the clear difference between the three culture batches. (**A**) Shoot length (cm). (**B**) Root length (cm). (**C**) Fresh shoot mass (g). (**D**) Fresh root mass (g). (**E**) Dry shoot mass (g). (**F**) Dry shoot mass (g). Error bars represent standard deviation. The circle refers to outliers. The “*” refers to extreme outliers.

**Figure 2:**
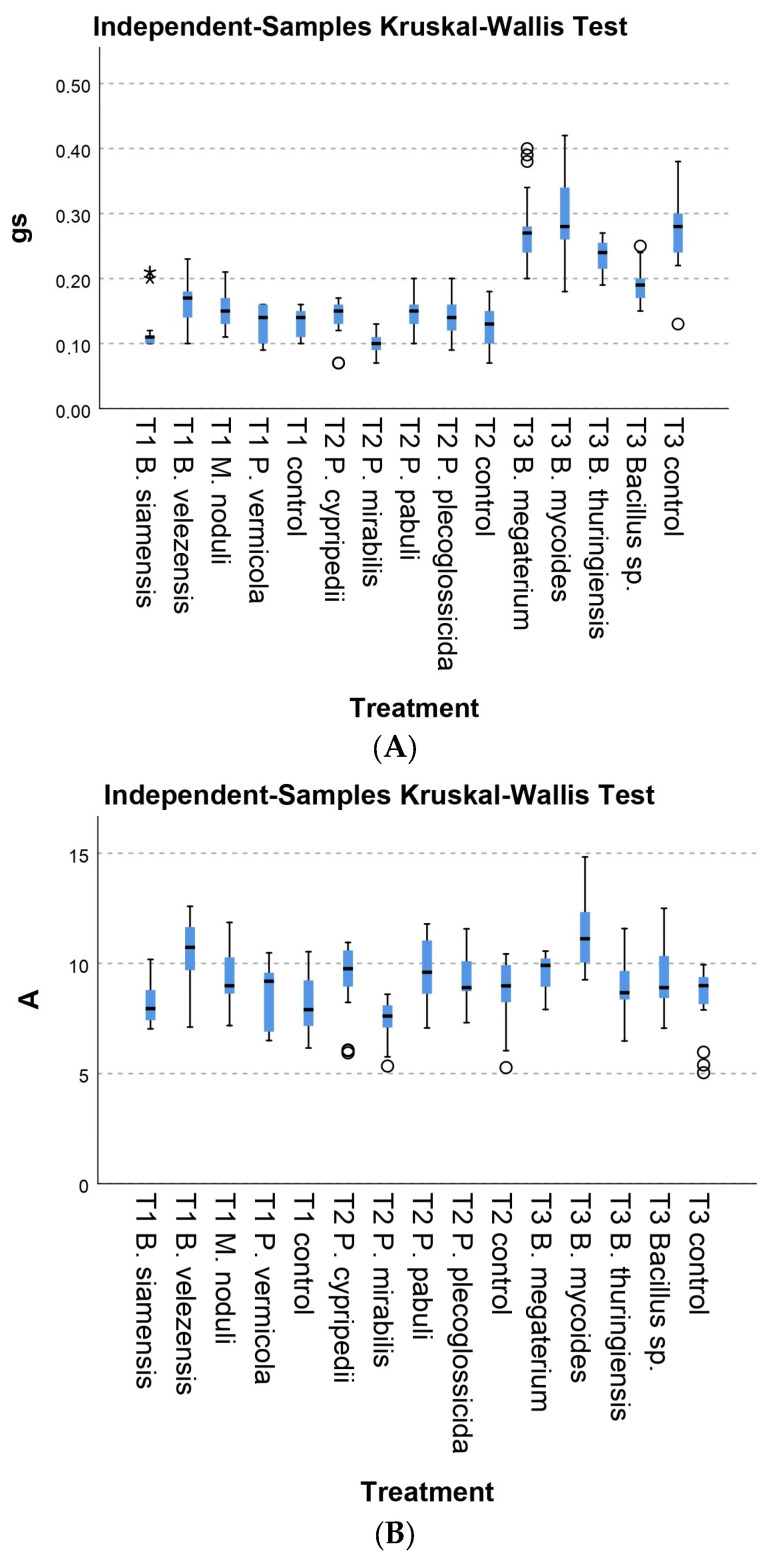
Kruskal–Wallis tests for the two physiological parameters studied. (**A**) Stomatal conductance. (**B**) Photosynthesis rate. Error bars represent standard deviation. The circle refers to outliers. The “*” refers to extreme outliers.

**Figure 3:**
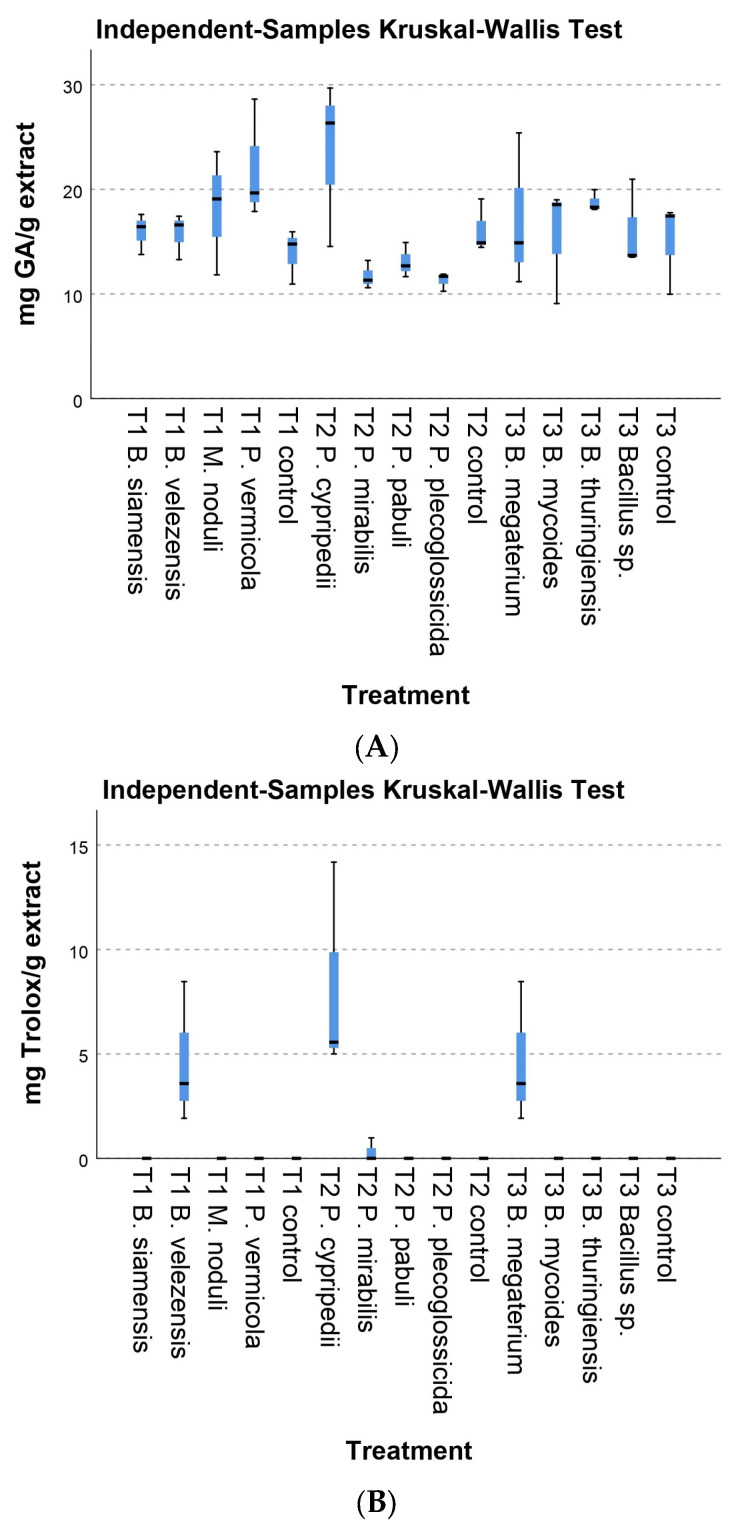
Kruskal–Wallis test for phenol and antioxidant content per gram of extract. (**A**) mg g^−1^ of Galic acid—phenol. (**B**) mg g^−1^ of Trolox—antioxidant. Error bars represent standard deviation.

Although the experimental conditions were repeated, designing the experiments in three separate sets lead to a significant variation within the controls groups. Therefore, while the Kruskal–Wallis box plots (Figure 1, Figure 2 and Figure 3) present all batches combined for simplification purposes, the statistical analysis was conducted independently for each set of experiments.

#### 3.2.1. Morphological Parameters

During the first round of trials, the treatments of *P. vermicola* 17, *M. noduli* 19, *B. siamensis* 1SEF, and *B. velezensis* 4PIN strains did not show significant results in aerial and root length compared to the control. However, the treatments of *M. noduli* 19, *B. siamensis* 1SEF, and *B. velezensis* 4PIN showed significant differences compared to the control aerial and root dry weight. *B. siamensis* 1SEF increased the shoot dry weight 32% more than the respective control (*p* = 0.019). On the other hand, tomatoes that received *B. velezensis* 4PIN inoculum showed a 33% increase in aerial dry weight versus the control (*p* = 0.044). Finally, in terms of root dry weights, the treatment with *B. siamensis* 1SEF represented an increase of 46% (*p* = 0.000), and the treatment with *B. velezensis* 4PIN presented an increase of 38% (*p* = 0.000) concerning the control without bacterial inoculum and *M. noduli* 19, presenting significantly higher values in both fresh weight (*p* = 0.000) and dry weight (*p* = 0.000) (Figure 1). 

The second assay showed much more promising results since significantly higher values were observed in the six variables studied (Figure 1). For aerial length, *P. plecoglossicida* 16 and *P. cypripedii* 32 strains stood out with significance values adjusted by the Bonferroni correction of *p* = 0.042 and *p* = 0.046, respectively. These strains significantly increased the aerial length compared to the control group. However, no significant differences were observed for strains *P. mirabilis* 27 and *P. pabuli* 47, with adjusted *p* values of 0.149 and 0.71, respectively. Despite this, the mean length was like that of the two previous strains, with this lack of differences being due to the variance in the treatments. Although the increase in size was subtle, with only a 2 cm difference in mean values of aerial length, which could suggest a potential for these strains to favor plant development. This was confirmed in root length, where all strains showed significant differences when compared with the control. These adjusted differences were *p* = 0.001, *p* = 0.000, *p* = 0.001, and *p* = 0.032 for strains 16, 27, 32, and 47, respectively. Inoculation with *P. mirabilis* 27 resulted in a root length increase of over 50% compared to the control, rising from an average of 28 cm to 43 cm (*p* = 0.037).

Regarding aerial and root weights, fresh and dry measurements revealed significant differences with the control for most treatments. The only strain that did not exhibit these differences was *P. pabuli* 47. In most treatments, aerial weight increased by more than 30% in fresh weight and over 40% in dry weight. Although strain *P. pabuli* 47 showed higher weights than the control, the 27% increase in dry weight was not statistically significant due to high variability (*p* = 0.198). In terms of root weight, increases exceeded 35% compared with the control in fresh weight, in plants inoculated with *P. plecoglossicida* 16, which exhibited a root fresh weight 55% higher than the control. The analysis of the remaining *Bacillus* strains was conducted during the third round of trials (Figure 1). *Bacillus* sp. 6AB and *B. megaterium* 8AB4 strains showed significant differences with control plants in shoot length (Figure 1A) and in fresh and dry aerial and root weight (Figure 1C–F). Plants inoculated with *B. megaterium* 8AB4 and *Bacillus* sp. 6AB showed an increase in aerial length of 22.43% (*p* = 0.000) and 17.87% (*p* = 0.001). *Bacillus* sp. 6AB and *B. megaterium* 8AB4 showed significant results concerning aerial fresh weight, 77.48% (*p* = 0.000) and 46.45% (*p* = 0.000), when compared with control plants. The fresh root weight only showed significant differences (*p* = 0.000 with *Bacillus* sp. 6AB strain), with a 62.26% increase.

On the other hand, the increase in aerial dry weights was 60.50% higher for B. megaterium 8AB4- (*p* = 0.000) and 77.06% for *Bacillus* sp. 6AB-inoculated plants (*p* = 0.000), following what had been reflected in both aerial length and aerial fresh weight, in which the increase values were significant. Dry root weight was 124% higher in plants treated with *Bacillus* sp. 6AB (*p* = 0.000) and 49% higher for the B. megaterium 8AB4 treatment (*p* = 0.002).

#### 3.2.2. Physiological Parameters

No significant differences were found between treatments and controls regarding the photosynthetic parameters analyzed: photosynthesis rate, stomatal conductance, maximum photochemical efficiency of photosystem II, or the photosynthetic yield index. This could indicate that the treatments did not alter photosynthetic processes under the experimental conditions. In contrast, the treatment with *B. velezensis* 4PIN presented a higher photosynthesis rate than the control plants (*p* = 0.000). These results among the biometric data indicate that its application improved plant development. Meanwhile, the treatment with strain *P. mirabilis* 27 showed significantly lower results compared to the other strains in its set (*p* = 0.000) and the control (*p* = 0.008) in two important parameters: stomatal conductance (Figure 2A) and photosynthesis rate (Figure 2B). These results indicate that treatment with this bacterium negatively affected the photosynthesis rate and the ability of leaves to regulate gas exchange with the environment, which could have hurt tomato plants.

Nevertheless, the morphological results showed an outstanding performance by *P. mirabilis* 27, exhibiting significantly higher values of root length and aerial and root weight than the control (Figure 1A,B). On the other hand, *B. mycoides* 1CRN also presented a higher photosynthesis rate than the control (Figure 2B). However, this did not translate into significant biometric data, perhaps because the pot size had limited plant growth, and the correlation between the physiological and morphological parameters needed to be clarified. 

Continuing with the different data from those pre-established in the hypotheses, there is a significant difference between the values of stomatal conductance presented by the *Bacillus* sp. 6AB treatment and the control (*p* = 0.000) (Figure 2A). The conductance in this treatment was lower than that observed in the control, which may be related to the timing of the measurements; these were taken at the end of the experiment rather than in a staggered manner throughout. Therefore, it cannot be affirmed that these lower values were stable over time. *Bacillus* sp. 6AB presented one of the best performances according to the biometric results.

Regarding the maximum photosystem II photochemical efficiency and photosynthetic yield index, no significant differences were observed between the treatments and the control (Figure 2B). This indicates that the treatments applied in this study did not affect the maximum photosynthetic capacity and the overall efficiency of light conversion to photosynthetic energy.

#### 3.2.3. Chemical Parameters

There were no differences in total phenols and photosynthetic pigments between the inoculated and control plants. The Kruskal–Wallis non-parametric test revealed differences in phenol content within the second group studied. However, these differences were not observed when compared with control plants but were significant between strains (Figure 3A). Specifically, significant differences were found between strains *P. plecoglossicida* 16 and *P. mirabilis* 27 compared to *P. cypripedii* 32, with *p* values of 0.014 and 0.018, respectively. However, when these values were adjusted via Bonferroni correction, they were *p* = 0.206 and *p* = 0.264, so there were no apparent differences. Regarding total antioxidant content (Figure 3B), the inoculated strains, *B. velezensis* 4PIN and *B. megaterium* 8AB4, showed a significantly higher antioxidant content than the controls (*p* = 0.050). However, the largest differences were for strains *P. cypripedii* 32 and *P. pabuli* 47, with unadjusted significance values *p* = 0.014 and *p* = 0.001, respectively, which when were adjusted *p* = 0.206 and *p* = 0.015.

#### 3.2.4. Nutritional Parameters

In the first set of the trial (Table 3), strains *P. vermicola* 17, *M. noduli* 19, *B. siamensis* 1SEF, and *B. velezensis* 4PIN presented higher percentages of the essential macronutrients N, P, and K when compared with the control. Meanwhile, the Ca content was higher in the *B. velezensis* treatment. The concentration of Mg increased in the shoot portion of plants treated inoculated with *P. vermicola* 17 and *B. velezensis* 4PIN. Regarding Na content, this micronutrient was only increased by *P. vermicola* 17. For Fe, most strains showed higher values in both the root and shoot zones than the control. This was repeated in the Mn and Cu micronutrients. Alternatively, Zn content was increased in the aerial part by *P. vermicola* 17, *B. siamensis* 1SEF, and *B. velezensis* 4PIN. Finally, in the case of B, *B. velezensis* presented higher concentrations than the control plants (Table 3). However, the results were entirely unexpected in the second set of strains (Table 4). The content of the macronutrients N, P, K, and Ca was higher in the control plants and in both the aerial and root parts, except for K with *P. plecoglossicida* 16- and *P. mirabilis* 27-inoculated plants. In addition, a higher content of the micronutrients Na, Mn, Cu, and B was also observed in the control plants.

Otherwise, the third trial set (Table 5) showed a significant presence of N in the aerial part of *Bacillus* sp. 6AB-inoculated plants. *B. megaterium* 8AB4-treated plants showed a higher percentage P in the aerial sections than the control plants. However, in the root zone, the highest amount was found in *B. mycoides* 1CRN-inoculated plants.

All strains showed lower K levels than the control, except in the case of *B. megaterium* for the shoot part, with similar amounts of this element. In the case of Ca, *B. megaterium* 8AB4 and *B. mycoides* 1CRN treatments showed higher percentages of this nutrient in the root and aerial zones than in the control. In the aerial zone, Mg was found in higher amounts in plants treated with *B. megaterium* 8AB4 strain. Na content increased in the aerial part of plants inoculated with *B. megaterium* 8AB4 and in the roots of those inoculated with *B. thuringiensis* 3GRA. In the study of Fe, an increase was observed in the root part of the treatments *Bacillus* sp. 6AB, *B. thuringiensis* 3GRA, and *B. mycoides* 1CRN, doubling, in the first case, the values of the control. About the aerial part, the treatment with strain *B. thuringiensis* 3GRA was the only one that presented values almost double those of the control. Mn and B did not improve with the treatments, being similar or slightly lower than the control, especially the root content for Mn and aerial for B. Cu presented remarkable values in roots of *B. megaterium* and *B. mycoides* inoculated plants. With the *B. thuringiensis* 3G treatment, there was only a slight improvement in the aerial section. In Zn, there was a clear difference between *B. mycoides* and control in the root portion.

## 4. Discussion

In this study, twelve characterized rhizobacterial strains with plant-growth-promoting characteristics were selected to evaluate their plant-growth-promoting effect on tomato (*Solanum lycopersicum*) plants. The treated plants with these rhizobacteria presented a larger root volume than the controls, which could be justified by the production of IAA by these strains, stimulating root development, especially in lateral roots and root hairs. Despite this larger root volume, longitudinal root development did not show a significant increase in all the treatments applied, as it could have been limited by the size of the pot and the experiment length itself, causing a restrictive effect on growth due to the lack of more area and substrate. The effectiveness of using PGPR to promote growth in tomato plants has been exemplified in a report by Khan et al. [16]. The *Bacillus* strains increased root volume, area, mean root diameter, and root length, a similar result found in these experiments, negatively influenced by the used pots. Other *Bacillus* strains, *B. subtilis* (FMCH002), and *B. lichenifromis* (FMCH001) were able to promote plant development by increasing root growth and both fresh and dry weights of the aerial and root parts of tomato plants after inoculation [56]. Similar results were reported with other *Bacillus* strains isolated from the rhizosphere of tomato plants, such as *B. thuringiensis*, *B. cereus*, *B. pacificus*, and *B. aerius*, improving biometric parameters of tomato plants inoculated under greenhouse conditions (root length and area, fresh and dry aerial and root weights) [57]. However, comparing the overall results with the data presented in this paper, inoculating non-native *Bacillus* strains (*Bacillus* sp. 6AB, *B. siamensis* 1SEF, *B. velezensis 4PIN*, and *B. megaterium* 8AB4) on tomato plants from different ecosystems deliver similar results in plant development. In fact, *B. thuringiensis* strain RZ2MS9 applied to tomato increased lateral root development because of its good IAA production [41]. Islam et al. [58] also found that inoculating the *B. siamensis* on tomato cultivars improved the biometric parameters relative to the control.

Previous studies using the *P. mirabilis* BETS2 strain showed a significant improvement in shoot and root growth in tomato and chili plants. This study demonstrates that selecting the strains used in the experiments by their plant growth promotion properties (siderophores production, IAA, phosphate solubilization, etc.) is essential [40]. Another report on the genus *Pantoea* [59] confirmed the benefits of inoculation with various strains on rice cultivars by increasing plant size, root length, and dry weight. Previously, Gholamalizadeh et al. [60] and Tahir et al. [61] had obtained similar results on rice and wheat. Regarding the *Paenibacillus* genus, Liu et al. [62] obtained significant results on tomato growth promotion. The strains *Paenibacillus* sp. SZ-15, *Paenibacillus* sp. BJ-4, *Paenibacillus* sp. SZ-10, *Paenibacillus* sp. SZ-14, *Paenibacillus* sp. YB-10, and *Paenibacillus* sp. WF-6 were exceptional, fixing nitrogen and producing IAA as plant growth promotion mechanisms. Eventually, the *Pseudomonas plecoglossicida* strain has been characterized as a phosphorus solubilizer [63,64]. The research highlights the beneficial impact of specific attributes on growth in wheat, maize, chickpea, and pigeon peas. All these results confirm those obtained in these experiments and can also be attributed to the mechanisms characterized in the strains, such as the production of phytohormones (IAA), solubilization, and nutrient mobilization (phosphorus, potassium, and nitrogen) [65].

The strains used in this study *B. megaterium* 8AB4, *Bacillus* sp. 6AB, *B. velezensis* 4PIN, *B. siamensis* 1SEF, *P. mirabilis* 27, and *P. plecoglossicida* 16 demonstrated significant IAA production with values between 2.5 and 21 μg mL^−1^. Additionally, several strains exhibited notable nutrient solubilization activity, including phosphorus (P) and potassium (K) solubilization, further promoting nutrient availability to the plants. Although *B. siamensis* 1SEF was unable to solubilize K, it effectively mobilized phosphorus, enhancing nutrient uptake. Another crucial mechanism identified was nitrogen fixation. Strains such as *P. mirabilis* 27, *B. megaterium* 8AB4, *B. siamensis* 1SEF, *B. velezensis* 4PIN, and *P. cypripedii* 32 all demonstrated nitrogen-fixing capabilities, which would have contributed to improved nitrogen availability for plant growth, further supporting the overall improvement in plant development. Thus, the potential of these strains to promote both aerial and root growth in tomato plants is evident. A vigorous plant with a well-developed and healthy root system is essential in agriculture, as this structure can significantly impact crop productivity and yield [66]. Moreover, as has been emphasized, the application of PGPR as a biofertilizer could be a promising alternative to traditional fertilizers and other conventional inputs, as many studies have already shown [67].

Many studies suggest a photosynthesis improvement after bacterial inoculation, as shown after *B. mycoides* strain 1CRN inoculation, although this did not translate into significant results in the biometric section [19]. Nevertheless, as in the case of *P. mirabilis* 27, the effect was the opposite, exhibiting a lower stomatal conductance and photosynthesis rate. The stress generated by substrate limitation and pot size could cause these negative data at the physiological level of the plant.

On the other hand, chemical parameters showed significant differences in the antioxidant levels of some treatments (*B. velezensis* 4PIN, *B. megaterium* 8AB4, *P. pabuli* 47, and *P. cypripedii* 32). Given the role of antioxidants in helping plants manage abiotic and biotic stresses [68], these variations may suggest potential abiotic stress under growing conditions. The role of antioxidants is safeguarding plants from oxidative stress, which results from an imbalance between the production of reactive oxygen species (ROS) and the plant’s ability to neutralize them [69]. Since ROS can damage cell membranes, proteins, and DNA, leading to cellular degradation and accelerated aging [70], it is plausible that the presence of antioxidants, such as vitamin C, vitamin E, or glutathione, could aid in neutralizing ROS and preserving cellular integrity [71]. Moreover, phenolic compounds, a group of secondary metabolites commonly found in plants, exhibit antioxidant properties that could further contribute to protection against oxidative stress. Although this study does not present direct evidence linking specific antioxidant levels to stress mitigation, it is something to explore in future research. Further studies could help clarify whether these antioxidant variations correspond with enhanced plant stress resilience and examine how these rhizobacteria may influence antioxidant production. In addition, these secondary metabolites can act as defense compounds, as some plants produce phenols in response to threats such as pathogens or herbivore attacks [72]. However, phenols may also be involved in intercellular communication and regulating plant growth and development [73]. Photosynthetic pigments also have multiple functions in plants. Chlorophylls absorb photons in the visible spectrum region and transfer energy through a series of reactions leading to the production of carbohydrates and oxygen [74]. Carotenoids, in addition to their role in capturing light energy for photosynthesis, especially in regions of the spectrum not absorbed by chlorophylls [75], are antioxidants that protect plant chloroplasts from damage caused by ultraviolet light and ROS. They also play a role in phytohormone synthesis, consequently regulating plants development and response to environmental factors [76]. Although no major differences were detected for the parameters studied, in this context, it can be hypothesized that plants treated with these strains may experience certain stresses, potentially leading to an increase in antioxidant production. It is important to emphasize that this assumption is purely an initial speculation, as more detailed and exhaustive investigations are required to determine the relationship between the bacterial strain and the plant.

Macronutrients levels such as N, P, and K showed differences, either in the aerial or root parts of the plants, when compared with control plants when specific strains were inoculated (*B. siamensis* 1SEF, *B. velezensis* 4PIN, *P. vermicola* 17, *P. plecoglossicida*, *Bacillus* sp. 6AB, *Bacillus* sp. 6AB, *B. megaterium* 8AB, and *B. mycoides* 1CRN). These data may be justified by the activation of direct mechanisms of P and K mobilization and solubilization as well as nitrogen fixation [10,11,12]. No significant differences were observed in general for Ca, Mg, and Na, while for Fe, Cu, and Zn, values varied between parts of the plant analyzed and the strains used. According to Montesdeoca-Flores et al. [77], these macro- and micronutrients are essential for plant development due to their beneficial effects. In fact, the presence of Fe is correlated with the activity of the siderophores produced by rhizobacteria, whose action allows for improving the assimilation and accumulation in roots of these metal by the plant [20,21]. Moreover, in these experiments with specific strains the Fe content in the roots increased by more than 1000 mg kg^−1^. This increase was not observed in the aerial part. However, due to its relatively immobile nature, this element tends to accumulate in the root [78]. In contrast, Zn, being a mobile element, accumulated in the aerial part and not in the root, exhibiting values more than double those of the control.

Although not all treatments resulted in improved plant nutrition, these findings in combination with the biometric and physiological parameters, support the beneficial effects of inoculation with these rhizobacteria. The plant promotion achieved using these microorganisms opens a promising window to their use in agricultural production by improving soil health and the relationship between soil and crops. Considering the variability of the treatments applied and the plant–bacteria specificity, it is reasonable to think that their use as biofertilizers can have a positive impact on agriculture.

## 5. Conclusions

The present study demonstrates that the strains *P. plecoglossicida*, *P. mirabilis*, *P. cypripedii*, *Bacillus* sp., *B. siamensis*, *B. velezensis*, and *B. megaterium* positively influence the growth and development of tomato plants under controlled laboratory conditions. These findings suggest that rhizobacteria isolated from various ecosystems in the Canary Islands could serve as potential biofertilizers for economically important crops, such as tomatoes. However, for these strains to be effective in practical applications, evaluating their performance under field conditions is crucial, where complex interactions with soil, climate, and native crop microbiomes could strongly influence their efficacy. Future studies must prioritize these field trials to understand how these rhizobacteria act in dynamic agricultural systems. In particular, research should address whether soil composition, nutrient availability, and environmental stressors, such as salinity and drought, impact the survival and effectiveness of these strains in promoting plant growth. Additionally, it would be valuable to explore the compatibility of these rhizobacteria with native soil microbiomes and any potential interactions that may emerge over time.

In addition, further research should focus on isolating and characterizing additional rhizobacterial strains from diverse ecosystems, particularly in non-agricultural zones of the Canary Islands. Such environments may harbor novel strains with unique mechanisms for promoting plant growth and resilience. Integrating omics technologies and systems biology could provide comprehensive insights into the molecular interactions between plants and rhizobacteria, helping to identify specific genes or pathways responsible for stress tolerance and growth promotion. This approach would enable the selection of strains best suited for various agricultural contexts and environmental conditions.

Finally, it is vital to consider the ecological implications of introducing non-native rhizobacteria into agricultural systems. Future studies should include long-term assessments of how these biofertilizers affect nutrient cycling, microbial diversity, and the overall health of soil ecosystems. This is especially critical as shifts in climate make conditions more challenging for crops, underscoring the need for biofertilizers that increase productivity and support sustainable agricultural practices without disrupting local ecosystems.

## Figures and Tables

**Table 1:** Strains used in the plant growth promotion study with their corresponding biochemical characterization determining their selection. This includes, with “+” or “−”, the capacity or not to solubilize phosphorus and potassium and the capacity to fix nitrogen. “++” indicates high solubilization capacity. The capacity to synthesize indoleacetic acid in µg/mL and siderophores in PSU is also included.

Strain	Species	P Sol.	K Sol.	N_2_ Fix.	IAA μg mL^−1^	PSU (7 Days)
16	*Pseudomonas plecoglossicida*	++	+	−	2.53 ± 3.42	15.81 ± 9.78
17	*Providencia vermicola*	+	+	+	101.77 ± 4.44	28.2 ± 3.01
19	*Mitsuaria noduli*	−	+	+	36.43 ± 2.05	40.89 ± 7.22
27	*Proteus mirabilis*	+	+	+	5.29 ± 2.22	47.78 ± 10.97
32	*Pantoea cypripedii*	++	+	+	0 ± 0	58.76 ± 3.18
47	*Paenibacillus pabuli*	+	−	+	0 ± 0	70.48 ± 1.59
1SEF	*Bacillus siamensis*	+	−	+	13.83 ± 0.55	52.24 ± 13.94
4PIN	*Bacillus velezensis*	+	+	+	21.82 ± 0.38	56.99 ± 6.54
1CRN	*Bacillus mycoides*	+	+	+	12.88 ± 0.86	39.24 ± 6.08
6AB	*Bacillus* sp.	+	+	−	18.43 ± 0.48	51.05 ± 5.80
8AB4	*Bacillus megaterium*	+	+	+	14.89 ± 1.31	61.01 ± 3.72
3GRA	*Bacillus thuringiensis*	+	+	−	12.55 ± 0.06	57.03 ± 6.51

**Table 2:** Soil analysis used as a substrate for the plant growth promotion trial showing in order from left to right; pH, % organic matter, ppm P_2_O_5_, Ca meq/100 g, Mg meq/100 mg, Ca/Mg, K meq/100 g, Na meq/100 g, EC mS/cm, and % SAT.

pH	% MO	P_2_O_5_	Ca	Mg	Ca/Mg	K	Na	CE mS/cm	% SAT
7.2	1.0	21	10.2	3.7	2.7	1.0	1.5	0.99	42

**Table 3:** Nutritional parameters of the aerial and root portion of tomato plants of the first batch. Macronutrients calcium, magnesium, potassium, phosphorus, and nitrogen g kg^−1^ dry plant. Micronutrients iron, manganese, copper, zinc, boron, and sodium in mg kg^−1^ dry plant. Different superscripts indicate significant difference (*p* < 0.05).

Strain	g kg^−1^					mg kg^−1^					
	N	P	K	Ca	Mg	Na	Fe	Mn	Cu	Zn	B
Aerial portion											
Control 1	14.00 ^a^	2.32 ^a^	12.92 ^b^	17.70 ^c^	4.00 ^b^	2208 ^c^	716 ^a^	75 ^a^	0 ^a^	4 ^a^	23 ^d^
*P. vermicola*	16.20 ^b^	2.54 ^b^	16.52 ^d^	15.33 ^a^	4.19 ^c^	2869 ^d^	1227 ^d^	101 ^c^	0 ^a^	5 ^b^	19 ^b^
*M. noduli*	16.90 ^c^	2.69 ^c^	12.06 ^a^	16.10 ^ab^	3.77 ^a^	1433 ^a^	1309 ^e^	95 ^b^	0 ^a^	3 ^a^	16 ^a^
*B. siamensis*	16.30 ^b^	2.77 ^c^	13.58 ^c^	15.67 ^ab^	3.83 ^a^	1652 ^b^	1116 ^b^	94 ^b^	33 ^c^	8 ^c^	20 ^c^
*B. velezensis*	16.80 ^c^	2.99 ^d^	12.94 ^b^	16.31 ^b^	4.11 ^c^	1536 ^a^	1201 ^c^	93 ^b^	6 ^b^	10 ^d^	25 ^e^
Root portion											
Control 1	18.80 ^b^	2.48 ^a^	24.75 ^b^	6.42 ^c^	9.30 ^b^	11,100 ^d^	3272 ^b^	466 ^b^	4 ^b^	27 ^c^	0 ^a^
*P. vermicola*	20.46 ^c^	2.59 ^a^	24.55 ^ab^	6.08 ^b^	9.15 ^b^	11,139 ^d^	3585 ^c^	579 ^d^	37 ^d^	17 ^a^	0 ^a^
*M. noduli*	16.10 ^a^	2.83 ^b^	23.93 ^a^	6.34 ^bc^	7.46 ^a^	8374 ^a^	3909 ^d^	526 ^c^	3 ^b^	23 ^b^	0 ^a^
*B. siamensis*	18.70 ^b^	2.82 ^b^	25.15 ^b^	5.77 ^a^	7.81 ^a^	9809 ^c^	3809 ^d^	646 ^e^	27 ^c^	18 ^a^	0 ^a^
*B. velezensis*	18.10 ^b^	2.83 ^b^	24.64 ^ab^	6.86 ^d^	7.69 ^a^	8863 ^b^	3040 ^a^	358 ^a^	2 ^a^	22 ^b^	0 ^a^

**Table 4:** Nutritional parameters of the aerial and root portion of tomato plants of the second batch. Macronutrients calcium, magnesium, potassium, phosphorus, and nitrogen g kg^−1^ dry plant. Micronutrients iron, manganese, copper, zinc, boron, and sodium in mg kg^−1^ dry plant. Different superscripts indicate significant difference (*p* < 0.05).

Strain	g kg^−1^					mg kg^−1^					
	N	P	K	Ca	Mg	Na	Fe	Mn	Cu	Zn	B
Aerial portion											
Control 2	18.90 ^d^	2.85 ^c^	15.95 ^b^	19.19 ^d^	3.95 ^a^	1982 ^e^	593 ^b^	86 ^e^	0 ^a^	7 ^a^	22 ^d^
*P. plecoglossicida*	14.90 ^b^	2.67 ^ab^	17.75 ^c^	15.68 ^a^	3.94 ^a^	1628 ^c^	350 ^a^	50 ^a^	0 ^a^	12 ^c^	16 ^ab^
*P. mirabilis*	14.10 ^a^	2.75 ^bc^	19.90 ^d^	16.13 ^ab^	3.94 ^a^	1710 ^d^	651 ^c^	73 ^c^	0 ^a^	18 ^d^	16 ^b^
*P. cypripedii*	15.07 ^c^	2.55 ^a^	14.53 ^a^	16.68 ^b^	3.84 ^a^	1156 ^a^	764 ^d^	80 ^d^	0 ^a^	7 ^ab^	14 ^a^
*P. pabuli*	14.99 ^bc^	2.62 ^ab^	16.02 ^b^	17.44 ^c^	3.83 ^a^	1347 ^b^	381 ^a^	65 ^b^	0 ^a^	8 ^b^	19 ^c^
Root portion											
Control 2	18.97 ^d^	3.20 ^d^	27.12 ^e^	7.33 ^c^	9.82 ^c^	10,980 ^e^	3989 ^c^	795 ^e^	11 ^d^	38 ^d^	0 ^a^
*P. plecoglossicida*	15.47 ^a^	1.56 ^a^	16.18 ^a^	5.84 ^b^	5.06 ^a^	4481 ^a^	3214 ^b^	261 ^a^	0 ^a^	13 ^a^	0 ^a^
*P. mirabilis*	16.26 ^b^	1.98 ^b^	20.27 ^c^	7.22 ^c^	6.56 ^b^	6641 ^c^	5050 ^d^	424 ^c^	2 ^b^	39 ^d^	0 ^a^
*P. cypripedii*	17.02 ^c^	1.91 ^b^	18.28 ^b^	5.07 ^a^	5.18 ^a^	5515 ^b^	2860 ^a^	346 ^b^	8 ^c^	19 ^b^	0 ^a^
*P. pabuli*	16.21 ^b^	2.18 ^c^	23.41 ^d^	7.30 ^c^	11.04 ^d^	7539 ^d^	5426 ^e^	453 ^d^	1 ^a^	22 ^c^	0 ^a^

**Table 5:** Nutritional parameters of the aerial and root portion of tomato plants of the third batch. Macronutrients calcium, magnesium, potassium, phosphorus, and nitrogen g kg^−1^ dry plant. Micronutrients iron, manganese, copper, zinc, boron, and sodium in mg kg^−1^ dry plant. Different superscripts indicate significant difference (*p* < 0.05).

Strain	g kg^−1^					mg kg^−1^					
	N	P	K	Ca	Mg	Na	Fe	Mn	Cu	Zn	B
Aerial portion											
Control 3	18.09 ^c^	1.82 ^d^	15.58 ^d^	15.67 ^d^	2.85 ^d^	1784 ^d^	331 ^d^	199 ^e^	0 ^a^	5 ^d^	24 ^e^
*Bacillus* sp.	20.16 ^d^	1.70 ^c^	15.12 ^c^	13.15 ^c^	2.68 ^c^	1625 ^c^	222 ^c^	113 ^b^	0 ^a^	7 ^e^	14 ^c^
*B. mycoides*	15.40 ^b^	1.27 ^b^	10.14 ^b^	10.17 ^a^	1.83 ^a^	963 ^a^	120 ^a^	92 ^a^	0 ^a^	1 ^b^	11 ^a^
*B. thuringiensis*	15.75 ^b^	1.13 ^a^	8.22 ^a^	12.07 ^b^	2.11 ^b^	1269 ^b^	511 ^e^	126 ^d^	3 ^b^	0 ^a^	12 ^b^
*B. megaterium*	14.81 ^a^	2.12 ^e^	15.73 ^d^	17.04 ^e^	3.14 ^e^	1887 ^e^	150 ^b^	151 ^d^	0 ^a^	3 ^c^	23 ^d^
Root portion											
Control 3	25.20 ^d^	2.67 ^c^	38.99 ^d^	4.11 ^a^	8.35 ^c^	13,346 ^c^	1880 ^b^	1549 ^e^	4 ^b^	44 ^c^	0 ^a^
*Bacillus* sp.	19.53 ^a^	1.22 ^a^	16.27 ^a^	5.40 ^c^	4.80 ^a^	5244 ^a^	4387 ^d^	659 ^a^	1 ^a^	17 ^a^	0 ^a^
*B. mycoides*	23.80 ^c^	3.44 ^d^	36.77 ^c^	6.93 ^e^	7.68 ^b^	12,171 ^b^	2014 ^c^	1192 ^d^	75 ^d^	78 ^d^	0 ^a^
*B. thuringiensis*	21.10 ^b^	2.33 ^b^	34.56 ^b^	6.25 ^d^	8.35 ^c^	13,808 ^d^	2034 ^c^	700 ^b^	5 ^b^	37 ^b^	0 ^a^
*B. megaterium*	22.40 ^bc^	2.80 ^c^	35.00 ^b^	5.12 ^b^	8.43 ^c^	13,047 ^c^	1715 ^a^	945 ^c^	18 ^c^	45 ^c^	0 ^a^

## Data Availability

The data used to support the findings of this study are included within the article.
